# Effect of Tumor Necrosis Factor Inhibitor Therapy on Osteoclasts Precursors in Rheumatoid Arthritis

**DOI:** 10.1155/2017/2690402

**Published:** 2017-02-13

**Authors:** Inês P. Perpétuo, Joana Caetano-Lopes, Ana Maria Rodrigues, Raquel Campanilho-Marques, Cristina Ponte, Helena Canhão, Mari Ainola, João E. Fonseca

**Affiliations:** ^1^Rheumatology Research Unit, Instituto de Medicina Molecular, Faculdade de Medicina, Universidade de Lisboa, Lisboa, Portugal; ^2^Rheumatology Department, Hospital de Santa Maria, Centro Hospitalar Lisboa Norte, EPE, Lisbon Academic Medical Centre, Lisboa, Portugal; ^3^EpiDoC Unit, CEDOC, NOVA Medical School, Universidade Nova de Lisboa, Lisboa, Portugal; ^4^Musculoskeletal Diseases and Inflammation Research Group, Biomedicum Helsinki 1, Faculty of Medicine, Institute of Clinical Medicine, University of Helsinki, Helsinki, Finland

## Abstract

*Objective*. Tumor necrosis factor (TNF) increases circulating osteoclast (OC) precursors numbers by promoting their proliferation and differentiation. The aim of this study was to assess the effect of TNF inhibitors (TNFi) on the differentiation and activity of OC in rheumatoid arthritis (RA) patients.* Methods.* Seventeen RA patients treated with TNFi were analyzed at baseline and after a minimum follow-up period of 6 months. Blood samples were collected to assess receptor activator of nuclear factor kappa-B ligand (RANKL) surface expression on circulating leukocytes and frequency and phenotype of monocyte subpopulations. Quantification of serum levels of bone turnover markers, in vitro OC differentiation assays, and qRT-PCR for OC specific genes was performed.* Results*. After TNFi therapy, patients had reduced RANKL surface expression in B-lymphocytes and the frequency of circulating classical CD14^bright^CD16^−^ monocytes was decreased. Serum levels of sRANKL, sRANKL/OPG ratio, and CTX-I were reduced in RA patients after TNFi treatment. Moreover, after exposure to TNFi, osteoclast differentiation and activity were decreased, as well as the expression of TRAF6 and cathepsin K.* Conclusion*. We propose that TNFi arrests bone loss and erosion, through two pathways: direct reduction of osteoclast precursor numbers and inhibition of intracellular signaling pathways acting through TRAF6.

## 1. Introduction

Rheumatoid arthritis (RA) is a chronic inflammatory disease characterized by systemic inflammation, bone erosion, and secondary osteoporosis [1].

The immune and skeletal systems have several regulatory factors in common and immune system cells have a profound influence on bone metabolism, particularly in the context of chronic inflammatory diseases. Receptor activator of nuclear factor-*κ*B ligand is present on osteoblasts' surface but is also expressed by activated immune cells, both in its membrane form and as a soluble molecule [2]. Tumor necrosis factor (TNF) increases the trafficking of immune system cells that efflux from bone marrow and peripheral blood into secondary lymphatic organs and sites of inflammation and is abundantly found in rheumatoid joints [3]. TNF, together with other cytokines, acts synergistically with the RANK-RANKL system [3, 4], further enhancing osteoclast (OC) differentiation from its circulatory precursors (monocytes) and contributing to bone resorption [2, 5]. It also increases the number of circulating OC precursors and the proinflammatory cytokine levels in RA patients. These effects are achieved with low levels of circulating TNF and thus TNF quantification is frequently unreliable in RA patients [6–8]. Of interest, TNF inhibitors (TNFi) have a beneficial effect in delaying radiographic damage in RA patients, even in the absence of clinical improvement, suggesting a specific effect of TNF inhibition, independent of inflammation control [9]. Whether this specific effect of TNFi in preventing bone damage in fact occurs independently of the overall inflammatory burden and whether it occurs because of reduced OC number and/or function are still unclear.

Our hypothesis was that, in RA patients, TNFi decrease the OC circulating precursors' differentiation potential and activity. Thus, the aim of this study was to assess the effect of TNFi in the differentiation and activity of OC precursors in a cohort of RA patients, evaluating also the correlation between clinical manifestations of inflammation and OC related parameters.

## 2. Patients and Methods

### 2.1. Patients

Patients with RA fulfilling the 2010 American College of Rheumatology/European League Against Rheumatism criteria [10] were recruited from the Rheumatology Department, Hospital de Santa Maria, Lisbon Academic Medical Centre, Portugal. All RA patients included were TNFi naïve and were followed up during a minimum of 6 months after starting TNFi therapy. Information regarding patients' demographics, duration of symptoms, erythrocyte sedimentation rate (ESR), C-reactive protein (CRP), tender and swollen joints counts, presence of erosion, presence of rheumatoid factor (RF), and presence of anticitrullinated protein antibodies (ACPA) was collected. Disease activity score (DAS28-CRP) was evaluated, as well as the Health Assessment Questionnaire (HAQ) [11].

Heparinized blood and serum samples were analyzed in baseline and follow-up samples after TNFi treatment approximately 6 months later. Whole blood samples were taken for flow cytometry and for isolation of peripheral blood mononuclear cells (PBMCs). Samples were stored at the Biobanco-IMM, Lisbon Academic Medical Center, Lisbon, Portugal. Patients were managed with the standard practice and all participants gave their informed consent. The study was approved by the local ethics committee and was conducted in accordance with the Declaration of Helsinki as amended in Brazil (2013).

### 2.2. Flow Cytometry

Identification of B- and T-cells and granulocytes in peripheral blood, RANKL surface expression, and immunophenotyping of monocytes in the PBMC samples were performed using matched combinations of anti-human murine mAbs as previously described [12]. Heparinized whole blood was used for flow cytometry and absolute cell counts were calculated from differential leukocyte count determined for all participants. Mononuclear cells were isolated from freshly drawn peripheral blood using density gradient centrifugation with Histopaque®-1077 (Sigma-Aldrich). Subpopulations of monocytes were identified based on the surface expression of CD14 and CD16 [13]. Median fluorescence intensity (MFI) was calculated based only on positive cells as determined by isotype control gating. FlowJo software (Tree Star, Stanford University) was used for analyzing flow cytometry data.

### 2.3. Bone Turnover Markers and Bone Metabolism Proteins Detection in the Serum

Carboxyterminal type I collagen cross links (CTX-I) for bone degradation products, human type I procollagen amino terminal propeptide (P1NP, Sunred Biological Technology) for bone formation, sclerostin (SOST), osteoprotegerin (OPG), Dickkopf-related protein-1 (DKK1), and soluble RANKL (ampli-sRANKL, Biomedica Gruppe) were analyzed with enzyme-linked immunosorbent assay [14] in serum samples according to the manufacturer's instructions.

### 2.4. PBMC Isolation and Cell Culture

PBMCs were isolated by density gradient centrifugation and plated in 96-well culture plates at a density of 7.0 × 10^5^ cells/well as described previously [12]. PBMCs were left overnight for OC precursors to adhere on bone slices and were further cultured for 21 days with macrophage-colony stimulating factor (M-CSF, 25 ng/mL, Peprotech), sRANKL (50 ng/mL, Peprotech), dexamethasone (10 nM, Sigma-Aldrich), and transforming growth factor-*β* (TGF-*β*, 2.5 ng/mL, R&D Systems), as described by our group [12]. Adherent cells at day 1 and cells cultured on bone slices for 7, 14, and 21 days [15] were used for functional assays and gene expression.

### 2.5. Functional Assays

OCs were stained for tartrate-resistant acid phosphatase (TRAP) at days 7, 14, and 21 using the Acid Phosphate Leukocyte Kit (Sigma-Aldrich) according to the manufacturer's instructions. Multinuclear cells containing three or more nuclei [16, 17] were counted as TRAP positive OCs. After visualization, cells were removed from bone slices using sodium hypochlorite and stained with 0.1% toluidine blue for the measurement of resorbed area at days 7, 14, and 21 of culture [18]. Bone slices were photographed in an area of 1.25 mm^2^ with a bright field microscope (Leica DM2500, Leica). The number of TRAP stained OCs was counted at each time point and resorption pits were traced using ImageJ software (NIH, Bethesda, MD). The resorbed area was expressed in % of total area.

### 2.6. Gene Expression

RNA was extracted from cells cultured over bone slices at days 1, 7, 14, and 21 of culture using NZYol (NZYTech) and complementary (c)DNA was synthesized as described previously [12]. Genes that encode osteoclast proteins such as RANK, TNF-receptor associated factor-6 (TRAF6), Fos-related antigen-2 (FRA-2), a subunit of H^+^-dependent ATPase (ATP6V0D2), TRAP, and cathepsin K (CTSK) were studied by real-time quantitative PCR (RT-qPCR) using the DyNAmo™ Flash SYBR Green qPCR Kit (Thermo Scientific). Primers (Suppl. Table  1 in Supplementary Material available online at https://doi.org/10.1155/2017/2690402) were designed using the primer-BLAST software [19]. The results were normalized with the housekeeping gene ribosomal RNA 18s and the standard curve method was used to determine the efficiency of qPCR as described previously [20, 21].

### 2.7. Statistical Analysis

Statistical analysis was performed with SPSS Statistics 17.0 (IBM) and GraphPad Prism 5 (GraphPad Software Inc.). Categorical variables were expressed as frequencies and comparisons were tested using chi-square test. Continuous variables were expressed by median and interquartile range. Spearman's correlations were performed between the analyzed parameters and clinical variables (ESR, CRP, tender and swollen joint count, and DAS28). Baseline and follow-up values of each sample were compared using Wilcoxon's matched-pairs signed-rank test or paired *t*-test according to normal distribution. *p* value less than 0.05 was considered significant.

## 3. Results

### 3.1. Patient Background

Seventeen RA patients, evaluated before and after starting TNFi therapy, were included in this study. All patients were receiving methotrexate (10–20 mg weekly), 15 of whom were also under low dose prednisolone and 2 were additionally under bisphosphonates. These therapies had been introduced more than 6 months before TNFi was started and were stable over the study period. Patients were treated with one of four TNFi: one of the monoclonal antibodies (adalimumab, golimumab, or infliximab; 41%) or etanercept (59%). A blood sample was obtained before the start of TNFi and after at least 6 months of treatment. Thirteen patients (76%) were good responders to TNFi and 4 (24%) were moderate responders according to the EULAR response criteria [22]. Joint counts, ESR, CRP, DAS28, and HAQ were significantly decreased after TNFi therapy. The clinical and demographic characteristics of patients both at baseline and at follow-up are described in Table 1.

### 3.2. TNFi Treatment in RA Patients Decreases the Frequency of Circulating Osteoclast Precursors

After TNFi treatment, the frequency of the classical monocyte subpopulation (CD14^bright^CD16^−^) was decreased (*p* = 0.0065; Table 2) and that of the nonclassical subpopulation (CD14^dim^CD16^+^) was increased (*p* = 0.0005) [13]. No differences were identified in either CD51/CD61 (*α*_v_*β*_3_ integrin) or RANK surface expression. After statistical correction for multiple comparisons, only the increase in the nonclassical subpopulation remained significant.

RANKL surface staining was performed in CD66b^+^ neutrophils, CD3^+^ T-cells, and CD19^+^ B-cells (Table 3). No difference was found in the total number of circulating neutrophils and T- or B-cells after therapy. Although the frequency of RANKL^+^ neutrophils or T-cells was not significantly different after treatment, both frequency and absolute number of RANKL^+^ B-cells were higher after treatment (*p* = 0.0088 and 0.0029, resp.). However, B-cell RANKL surface expression was significantly decreased after treatment (*p* = 0.0401). When statistically corrected for multiple comparisons, the increase in RANKL^+^ B-cells remained significant.

### 3.3. The sRANKL/OPG Ratio and CTX-I Circulating Levels Are Reduced in RA Patients after TNFi Treatment

Circulating levels of sRANKL were significantly decreased after TNFi (*p* = 0.0085; Table 4), leading to decreased sRANKL/OPG ratio (*p* = 0.0031). We found no differences in the circulating levels of DKK1 or SOST. CTX-I and P1NP levels were lower in patients at 6 months of follow-up, when compared to patients at baseline (*p* = 0.0005 and 0.0252, resp.), and no difference was found in the CTX-I/P1NP ratio. After correcting for multiple comparisons, the differences in sRANKL/OPG and CTX-I after treatment remained significant.

### 3.4. Osteoclast Differentiation and Activity in RA Patients Are Decreased after TNFi Treatment due to Decreased TNF Intracellular Signaling and Cathepsin K Expression

**Figure 1:**
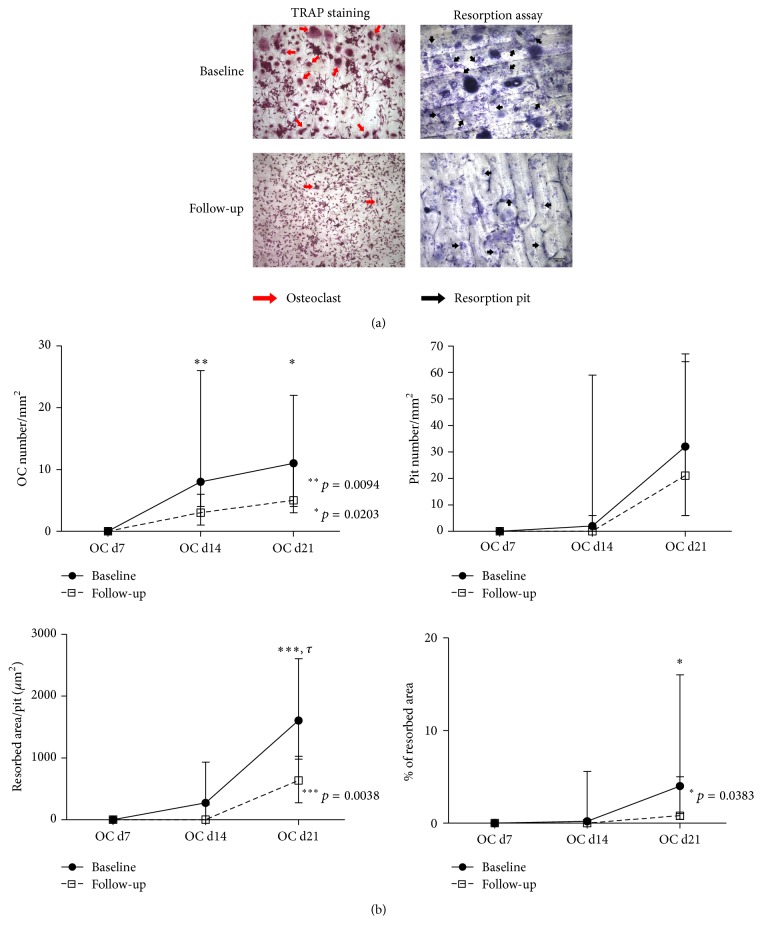
Functional assays of in vitro differentiated OC. (a) Representative images, at culture day 21, of adhering precursors stimulated with M-CSF, RANKL, dexamethasone, and TGF-*β* stained for TRAP, where the pit assay was performed. (b) OC number increased throughout time and, at culture days 14 and 21, patients at follow-up had significantly fewer osteoclasts than at baseline (*p* = 0.0094 and 0.0203, resp.). No differences were found in the number of resorption pits/mm^2^; patients at follow-up had significantly smaller pits at culture day 21 (resorbed area/pit, *p* = 0.0038) and significantly less resorbed area at culture day 21, when compared to their baseline (*p* = 0.0383). Dots represent median counts for each group at each time point and bars represent interquartile range. d: day; OC: osteoclast. Scale bars: 100 *μ*m; red arrows: osteoclasts; black arrows: resorption pits. *τ*: remained significant after adjusting for multiple comparisons.

Under stimulating conditions, adhering precursors from patients treated with TNFi formed fewer OCs than adhering precursors from patients at baseline (*p* = 0.0094 at culture day 14, *p* = 0.0203 at culture day 21; Figure 1).

Although the number of resorption pits was not significantly different before and after treatment, the area resorbed per pit was significantly reduced in cultures from patients at follow-up at culture day 21 (*p* = 0.0038), which resulted in significantly decreased total resorbed area (*p* = 0.0383). After statistical correction for multiple comparisons, only the differences in OC number at day 14 and the resorbed area per pit at day 21 remained significant.

**Figure 2:**
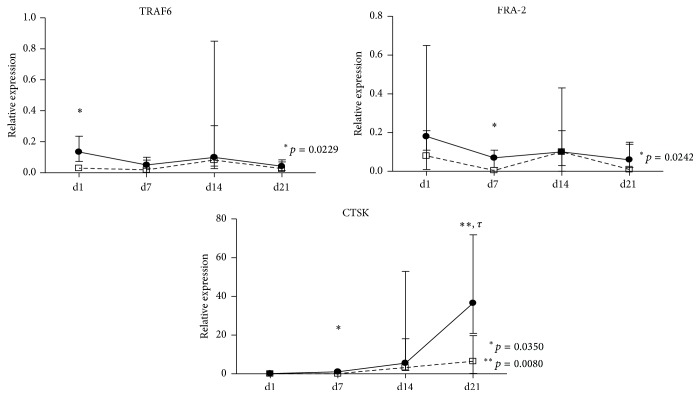
Gene expression profile of stimulated adhering precursors in culture for 21 days. At day 1, TRAF6 expression in patients at follow-up was significantly reduced (*p* = 0.0229). At day 7, both FRA-2 and CTSK expressions were significantly decreased (*p* = 0.0242 and 0.035, resp.). At day 21, patients at follow-up had significantly reduced expression when compared to patients at baseline (*p* = 0.008). Gene expression shown as a ratio to housekeeping expression (2(−ΔCT)/2(−ΔCT)). Dots in graphs represent median gene expression for each group at each time point and lines represent interquartile range [25–75]. d: day; TRAF6: gene encoding tumor necrosis factor receptor-associated factor-6; FRA-2: gene encoding Fos-related antigen-2; CTSK: gene encoding cathepsin K. *τ*: remained significant after adjusting for multiple comparisons.

Gene expression by RT-qPCR was performed for OC genes that are known to be important during the adhering precursors' differentiation and OC activity. At culture day 1, TRAF6 expression in patients at follow-up was significantly lower than in patients at baseline (*p* = 0.0229; Figure 2). At culture day 7, expression of both FRA-2 and CTSK was significantly decreased after TNFi treatment (*p* = 0.0242 and 0.0350, resp.). No differences were found in any of the studied genes at culture day 14, but at culture day 21 there was a significant decrease in CTSK expression in the differentiated OC from patients after treatment. This difference in CTSK expression remained significant after multiple comparisons adjustment.

No differences were found in any of the studied parameters when comparing monoclonal antibodies (adalimumab, infliximab, or golimumab) with the fusion protein etanercept (data not shown). No correlation was found between clinical or laboratorial inflammatory parameters for any of the studied variables.

## 4. Discussion

With this study, we aimed to test the effect of TNFi in the differentiation and activity of OC precursors in RA patients.

We have shown that RA patients treated with TNFi have reduced frequency of classic monocytes. We also found a decrease in the circulating levels of soluble RANKL and consequently a reduction in the sRANKL/OPG ratio after TNFi treatment. Although no differences in circulating levels of SOST or DKK1 were detected, serum CTX-I and P1NP levels were decreased after TNFi treatment, reflecting decreased bone turnover in these patients. Accordingly, we found that the ex vivo differentiation and resorptive activity of OC precursors from TNFi-treated patients were reduced, mainly due to early downregulation of TNF signaling proteins, such as TRAF6 or FRA-2, and to a later reduction of CTSK expression. Moreover, when comparing all studied parameters, we found no differences between the use of monoclonal antibodies (adalimumab, golimumab, and infliximab) and the fusion protein [23], suggesting that they have similar effects on OC precursors. Previous studies have compared the effects of different TNFi in disease activity, sRANKL/OPG ratio, and circulating leukocytes without finding significant differences [24, 25]. It has been previously reported that granulocyte numbers were reduced in circulation after 2 and 14 weeks of infliximab treatment [26]; however, this study identified granulocytes as CD16^+^ cells instead of CD66b^+^ cells. We found no significant differences in the frequency of neutrophils and T-lymphocytes or in RANKL surface expression in these cells, but we observed a significant increase in RANKL^+^ B-lymphocytes accompanied by a decrease in RANKL surface expression. There have been a number of studies addressing the effect of TNFi in RA patients' peripheral lymphocytes; however, there is no consensus among different reports, mainly due to sampling differences. In 2005, Toubi and colleagues have shown that infliximab decreased apoptosis in Tregs of RA patients [27]. Other studies showed that short in vitro exposure of PBMCs to infliximab or etanercept had no effect in peripheral lymphocyte apoptosis [28] or in synovial membrane biopsies [29]. It has previously been shown that RA patients under TNFi have increased number of T-regulatory cells and a reduced number of T-effector cells [30]. Other studies showed that in TNFi-treated RA patients there were no changes in T-regulatory cells frequency [24] or in the frequency of total T-cells, monocytes, or granulocytes and only a transient unspecified effect on B-cells [31]. To our knowledge, a comparative study of RANKL expression in RA patients before and after therapy with TNFi has never been published.

Three monocytes subpopulations, based on their expression of CD14 and CD16 surface markers, have been described in humans [13]. In RA patients, it has been shown that the intermediate subpopulation is increased when compared to healthy donors [13] and apoptosis of local and peripheral monocytes/macrophages was also increased after etanercept or infliximab treatment [29, 32]. Another study has shown no differences in CD14^dim^ or CD14^bright^ subpopulations after 4 months of infliximab therapy [26]. In our cohort, 6 months after TNFi therapy, patients showed decreased classic (CD14^bright^CD16^−^) and increased nonclassical (CD14^dim^CD16^+^) subpopulations. These changes in frequency were accompanied by a nonsignificant decrease in CD51/CD61 (*α*_v_*β*_3_ integrin) and RANK surface expression in all subsets. In accordance with our results, a recent study showed a reduction in classical monocyte subpopulation and an increase in the nonclassical subpopulation following infliximab therapy [33]. Moreover, Sprangers et al. observed that although nonclassical monocytes can also differentiate into OC, these cells have lower resorptive ability [34], which might explain why we did not observe bone resorption increase.

Patients under TNFi had reduced levels of sRANKL, sRANKL/OPG, CTX-I, and P1NP, suggesting a decrease in OC activity and a return to balanced coupling of bone resorption and bone formation. No differences were found in the circulating levels of DKK1 and SOST after TNFi treatment. Previous studies have shown discrepancies in the determination of these bone remodeling-associated proteins. Studies have found no differences in sRANKL or OPG serum levels after infliximab or etanercept [35, 36]. However, contradictory results have emerged regarding both OPG and sRANKL circulating levels after TNFi therapy [37, 38]. DKK1 and sclerostin have a direct effect on bone formation through interaction with the Wnt signaling pathway [39] but they have not been extensively studied in RA patients under TNFi. Previous reports have shown that etanercept has no effect on circulating levels of DKK1 but it increases sclerostin in circulation after treatment [35]. However, infliximab has been shown to decrease DKK1 levels in patients responding to therapy [40]. It has been previously shown that TNFi have a beneficial effect, reducing radiographic damage in RA patients, even in the absence of clinical improvement [9, 41]. Reports have described a decrease in CTX-I or urinary markers of bone resorption after TNFi therapy [35, 42]. However, some discrepancies have been found when studying bone formation markers. Studies with etanercept and infliximab showed no alteration in circulating P1NP levels after treatment [35, 42], while another study with etanercept showed reduced levels of urinary bone formation markers [43].

Although the classical monocyte subpopulation has been considered the OC precursor subset, all three subpopulations can differentiate in vitro into OC [44]. To understand the effect of TNFi in OC differentiation and function, we isolated PBMCs from RA patients before and after TNFi treatment and cultured them in vitro over bone slices. After TNFi treatment, we found a decrease in OC number and both in the total resorbed area and in the average resorbed area per pit. No differences in pit number and in the number of nuclei/OCs, aspects associated with OC activity, were identified [45]. These observations suggest that TNFi reduces the number and mobility of OCs.

Complex in vivo studies with animal models also showed that infliximab and etanercept reduced the bone resorbed area [46, 47] and etanercept decreased *α*_v_*β*_3_ integrin expression [48]. In a study similar to ours, Gengenbacher and colleagues studied RA patients under infliximab therapy for 6 months and observed decreased pit number after in vitro cell culture in OC differentiating conditions [36]. There have been reports that infliximab inhibits directly (in vitro) murine and human OC formation [49, 50]. Other authors show that although TNFi reduce the number of murine pre-OCs in vitro, there is no effect in the total number of formed OCs [51]. Another study has shown that infliximab directly inhibits OC formation in high density healthy PBMC cultures without any further stimuli [52]. Etanercept was also shown to inhibit in vitro OC formation induced by M-CSF and IL-23 from healthy subjects [53]. Controversially, Takita and colleagues cultured PBMCs from RA patients, exposing them to M-CSF, RANKL, and infliximab in vitro, and observed that infliximab increased bone resorption when compared to M-CSF and RANKL alone [54].

There is evidence that TNF contributes to expression of specific OC proteins and that it directly activates OC differentiation through cross activation of the NF-*κ*B pathway or c-Jun N-terminal kinase (JNK) signaling cascade [55]. We were interested in understanding the underlying mechanisms of reduced OC formation and bone resorption after TNFi, so we conducted gene expression assays and observed that OC precursors from RA patients after TNFi exposure had decreased expression of TRAF6 at culture day 1, followed by a reduction of FRA-2 and CTSK at day 7, and finally decreased expression of CTSK at culture day 21, when compared to patients before TNFi exposure. RANK/RANKL signaling cross-talks with TNF signaling, as RANK is a TNF-superfamily member [56]. Upon activation, both RANK and TNF activate cytoplasmic kinases and adaptor proteins, including TRAF6, which further activate FRA-2 [57]. FRA-2 is a protein that when associated with Fos and AP-1 promotes the transcription of OC differentiating genes, including CTSK [58]. In TNFi-treated patients, we have observed not only a decrease in serum CTX-I (cleaved by CTSK), but also a reduction in CTSK expression after adhering precursors differentiation in vitro, as well as a decline in the resorbed area/OC. This has previously been observed in a RA patient with concomitant pycnodysostosis, an autosomal recessive mutation in the cathepsin K gene characterized by absence of this enzyme. Osteoclasts from these patients form very small resorbing pits and do not release CTX-I into the culture media [59].

The main limitations of this work were the lack of healthy controls and the reduced number of patients and the diversity of TNF blockers studied, which we tried to overcome by studying the same patient before and after therapy.

Taken together with the results found in the literature, these findings suggest that TNFi decrease bone resorption, independently of the control of disease activity. We propose that this is due to the direct reduction of OC classical precursors and downregulation of intracellular signaling pathways involving TRAF6 resulting in a reduction of CTSK expression and consequent lack of OC motility. Further investigation of the signaling pathways involving TRAF6, such as the ASK1-TRAF6 interaction, is of clear interest in this context.

## Supplementary Material

Primers were designed using primer-BLAST software [19] and adhered to the following specifications: they had to be in an exon-exon junction, at annealing temperature of 60°C, with transcript under 150 bp.

## Figures and Tables

**Table 1:** Baseline and follow-up characteristics of patients.

		RA patients (*n* = 17)		*p*-value
	Baseline		Follow-up	
Age (years)		50 [38–63]		—
% Females		71%		—
Symptoms duration (years)		6 [3.5–9.5]		—
Rheumatoid factor (% positive)		71		—
ACPA (% positive)		53		—
Erosive (% y)		59		—
Treatment with NSAIDs (% y)		47		—
Treatment with DMARD (% y)		100		—
DMARD duration (months)		15 [3–51]		—
ESR (mm/h)	28 [18–48]		21 [13–26]	0.0257
CRP (mg/dl)	1.4 [0.7–2.0]		0.3 [0.04–0.8]	0.0018
Tender joint count	9 [4–14]		0 [0–2]	0.0005
Swollen joint count	7 [4–9]		0 [0-0]	0.0005
DAS28-CRP	5.6 [5.2–6.3]		2.9 [2.2–3.5]	<0.0001
HAQ	1.7 [0.8–2.0]		0.1 [0.0–1.0]	0.0059
TNFi duration (months)	—		6 [6–12]	—

Data is represented as median [Interquartile range] unless stated otherwise; *p*-value < 0.05 is considered significant; ACPA - anti-citrullinated protein antibodies; CRP – C-reactive protein; DAS – disease activity score; DMARDs – disease modifying antirheumatic drugs; ESR – erythrocyte sedimentation rate; HAQ - Health assessment questionnaire; NSAIDs - non-steroidal anti-inflammatory drugs; RA – rheumatoid arthritis; TNFi – tumor necrosis factor inhibitors; y – yes.

**Table 2:** Monocyte subpopulation frequency and osteoclastogenic marker expression.

	Baseline	Follow-up	*p* value
Classic (%)^a^	88 [82–89]	78 [70–83]	0.0065^*∗∗*^
Classic CD51/CD61 MFI	130 [119–148]	125 [111–137]	0.4258
Classic RANK MFI	133 [116–160]	122 [100–135]	0.1849

Intermediate (%)^a^	4.4 [2.4–5.6]	4.0 [2.1–7.1]	0.6013
Intermediate CD51/CD61 MFI	222 [139–400]	193 [146–240]	0.8203
Intermediate RANK MFI	197 [117–361]	188 [120–272]	0.9102

Nonclassic (%)^a^	5.7 [4.1–11]	14 [11.5–18.1]	0.0005^*∗∗∗*,†^
Nonclassic CD51/CD61 MFI	192 [80–290]	142 [127–167]	0.5703
Nonclassic RANK MFI	139 [122–157]	138 [126–146]	1.0000

Flow cytometry results are shown as median and interquartile range; ^a^gated on the monocyte subpopulation from peripheral blood mononuclear cells. RANK: receptor activator of nuclear factor-*κ*B; MFI: median fluorescence intensity (arbitrary units); ^*∗∗*^*p* < 0.01, ^*∗∗∗*^*p* < 0.001. ^†^Remained significant after correction for multiple comparisons.

**Table 3:** Whole blood cell distribution and RANKL expression.

	Baseline	Follow-up	*p* value
Neutrophils (%)^a^	82 [71–91]	90 [84–091]	0.2662
Neutrophils (×10^8^ cells/L)	12.7 [8.0–15.6]	9.6 [8.4–12.9]	0.2642
RANKL^+^ neutrophils (%)^b^	22 [3–41]	53 [21–77]	0.0856
RANKL^+^ neutrophils (×10^8^ cells/L)	1.5 [0.3–4.3]	5.9 [1.8–7.1]	0.1475
Neutrophil RANKL MFI	33.2 [25.5–44.9]	24.1 [21.7–28]	0.0830

T-cells (%)^c^	62 [58–74]	68 [52–72]	0.5265
T-cells (×10^8^ cells/L)	4.2 [2.4–5.2]	3.4 [2.4–11.7]	0.4131
RANKL^+^ T-cells (%)^b^	6.2 [0.8–24]	6.7 [4.6–15.7]	0.8984
RANKL^+^ T-cells (×10^8^ cells/L)	0.30 [0.03–1.03]	0.20 [0.16–0.69]	0.7646
T-cell RANKL MFI	49 [41–55]	32 [25–53]	0.2061

B-cells (%)^c^	7.3 [4.8–14]	9.2 [4.9–15.0]	0.7364
B-cells (×10^8^ cells/L)	0.40 [0.18–0.94]	0.44 [0.23–1.51]	0.9658
RANKL^+^ B-cells (%)	4.7 [2.0–6.7]	14 [3–28]	0.0088^*∗∗*^
RANKL^+^ B-cells (×10^8^ cells/L)^b^	0.02 [0.01–0.06]	0.06 [0.02–1.22]	0.0029^*∗∗*,†^
B-cell RANKL MFI	48 [38–80]	30 [25–63]	0.0401^*∗*^

Flow cytometry results are shown as median and interquartile range; ^a^gated on granulocytes from whole blood; ^b^gated on the correspondent parent gate (neutrophil, T- or B-cell); ^c^gated on the nongranulocyte cells from whole blood (also called the “monolymph” gate). RANKL: receptor activator of NF-*κβ* ligand; MFI: median fluorescence intensity (arbitrary units); ^*∗*^*p* < 0.05, ^*∗∗*^*p* < 0.01. ^†^Remained significant after correction for multiple comparisons.

**Table 4:** Serum levels of bone turnover markers and bone metabolism proteins.

	Baseline	Follow-up	*p* value
sRANKL (pmol/L)	0.32 [0.21–0.67]	0.18 [0.11–0.35]	0.0085^*∗∗*^
OPG (pmol/L)	4.34 [2.60–5.82]	4.22 [3.05–5.08]	0.7990
sRANKL/OPG	0.08 [0.04–0.17]	0.05 [0.03–0.07]	0.0031^*∗∗*,†^
DKK1 (pmol/L)	25.5 [18.1–43.3]	26.4 [21.9–31.7]	1.000
Sclerostin (pmol/L)	25.2 [16.94–33.8]	25.2 [19.2–29.3]	0.8577
CTX-I (ng/mL)	194.6 [176.6–430.7]	163.6 [152.1–173.9]	0.0005^*∗∗∗*,†^
P1NP (ng/mL)	55.7 [46.3–61.3]	45.8 [39.6–48.9]	0.0252^*∗*^
CTX/P1NP	3.36 [3.09–3.82]	3.71 [3.34–4.30]	0.5590

Enzyme-linked immunosorbent assay results are shown as median and interquartile range. sRANKL: soluble receptor activator of NF-*κβ* ligand; OPG: osteoprotegerin; DKK1: Dickkopf-related protein-1: CTX: carboxyterminal telopeptide of type I collagen; P1NP: total procollagen type 1 N-terminal propeptide; ^*∗*^*p* < 0.05, ^*∗∗*^*p* < 0.01, and ^*∗∗∗*^*p* < 0.001. ^†^Remained significant after correction for multiple comparisons.

## Additional Points

*Key Messages.* (i) TNFi decrease bone resorption through the direct reduction of OC precursor numbers. (ii) TNFi downregulate the intracellular signaling pathways involving TRAF6 resulting in a reduction of CTSK expression and consequent lack of OC motility.
