# Supplementary material for: Effect of Tumor Necrosis Factor Inhibitor Therapy on Osteoclasts Precursors in Rheumatoid Arthritis
Source: Biomed Res Int. 2017 Feb 13;2017:2690402. doi: 10.1155/2017/2690402 (PMC5327780; doi:10.1155/2017/2690402)
Supplement: Supplementary file 1 — Primers were designed using primer-BLAST software [19] and adhered to the following specifications: they had to be in an exon-exon junction, at annealing temperature of 60°C, with transcript under 150 bp. [file 2690402.f1.docx]

**Supplementary table 1 - Primers used for osteoclast gene expression**

| **Gene** | **Primer sequence** | **Annealing temperature** | **Transcript size** |
| --- | --- | --- | --- |
| RANK | Fw 5' - GAACATCATGGGACAGAGAAATC - 3'  Rev 5' - GGCAAGTAAACATGGGGTTC - 3' | 60ºC | 89bp |
| TRAF6 | Fw 5' - GCACTAGAACGAGCAAGTGAT - 3'  Rev 5' - GGCAGTTCCACCCACACTAT - 3' | 60ºC | 153bp |
| FRA-2 | Fw 5' - CAGCAGAAATTCCGGGTAGA - 3'  Rev 5' - CATGGAGGTGATCACTGTGG - 3' | 60ºC | 120bp |
| ATP6V2D0 | Fw 5' - CATTCTTGAGTTTGAGGCCG - 3'  Rev 5' - CCGTAATGATCCGCTACGTT - 3' | 60ºC | 186bp |
| TRAP | Fw 5' - CGGCCACGATCACAATCT - 3'  Rev 5' - GCTTTGAGGGGTCCATGA - 3' | 60ºC | 92bp |
| CTSK | Fw 5' - GCCAGACAACAGATTTCCATC - 3'  Rev 5' - CAGAGCAAAGCTCACCACAG - 3' | 60ºC | 75bp |
| 18s rRNA | Fw 5' - GGAGTATGGTTGCAAAGCTGA - 3'  Rev 5' - ATCTGTCAATCCTGTCCGTGT - 3' | 60ºC | 129bp |
